# Analysis of the Effect of the Elongation Operation on the Welding of Internal Metallurgical Discontinuities

**DOI:** 10.3390/ma16206738

**Published:** 2023-10-18

**Authors:** Grzegorz Banaszek, Teresa Bajor, Anna Kawałek, Marcin Knapiński

**Affiliations:** Faculty of Production Engineering and Materials Technology, Czestochowa University of Technology, 42-201 Czestochowa, Poland; grzegorz.banaszek@pcz.pl (G.B.); anna.kawalek@pcz.pl (A.K.); marcin.knapinski@pcz.pl (M.K.)

**Keywords:** magnesium alloy AZ91, physical modelling, forging, closure of discontinuities

## Abstract

This article discusses the results of our research into the effect of elongation on the welding of internal metallurgical discontinuities for two different geometrical shapes of a model feedstock of a selected magnesium alloy. Model discontinuities, specifically those of the metallurgical void type, were placed in various local zones of the modelled feedstock to check the influence of their location on their welding. The numerical modelling was carried out using the Forge^®^NxT2.1 application based on the finite element method. The results of the numerical tests were verified in laboratory conditions using the Gleeble simulator of metallurgical processes. Based on this research, it was found that the geometric shape of the feedstock material and the location of internal metallurgical discontinuities have a significant impact on the welding of discontinuities. The optimal values of the main process parameters of the elongation operation in flat dies were also determined for use in individual forging stages in order to eliminate internal metallurgical discontinuities. On the basis of the numerical studies carried out and their verification under laboratory conditions, it was concluded that a relative draft equal to 35% should be applied to weld the metallurgical discontinuities, which would result in a favorable hydrostatic pressure distribution within the discontinuities.

## 1. Introduction

The search for lightweight construction materials that are characterized by favorable strength parameters is a topical current area of interest among scientific circles, which invariably considers magnesium alloys to be among the most important of the group of materials mentioned above. Nowadays, since electric cars make up an increasing percentage of the manufactured assortment of cars, reducing the curb weight of cars and the consumption of energy or fuel is very important for manufacturers and consumers [1,2,3,4,5,6]. Magnesium alloys, as the lightest construction material exhibiting good heat dissipation and vibration damping, have been of interest to the automotive industry for over two decades [7,8,9,10,11,12,13,14,15,16]. However, a large number of products made of magnesium alloys are obtained in the processes of extrusion and stamping, and less often, they are obtained in the processes of rolling and forging, despite the fact that scientific research shows that forged products made of magnesium alloys have a homogeneous microstructure and better mechanical properties compared to the products made of cast alloys. Designing a forging technology is not easy and requires a holistic approach to the research problem. The high variability of shaping parameters, such as temperature, sequence of operations and treatments (e.g., material rotation and deformation with the use of dies during the forging elongation operation), draft presets, relative feed values, the rate of deformation, and the shape and dimensional parameters of the working surfaces of the dies, make it extremely difficult to obtain high-quality magnesium alloy forgings with a uniform microstructure throughout their volume. Only well-thought-out and correct implementation of the free forging process in flat and shaped dies of selected magnesium alloys allows us to obtain a product with satisfactory final properties [17,18]. An unquestionable advantage of using the elongation operation in the processes of shaping products is the possibility of closing internal metallurgical discontinuities arising at the stage of production of the input material [19,20,21,22,23,24,25,26,27]. Various methods of closing internal metallurgical discontinuities in the cross-section of forgings were studied and the results can be found in the works of [26,27,28,29,30], where it was shown that the closure of metallurgical discontinuities in deformed bars is affected by the main parameters of the forging process, such as draft, relative feed, feedstock temperature, and the shape and dimensions of the dies.

Conducting research on the impact of elongation operations on the closing metallurgical discontinuities in industrial conditions is ineffective from the point of view of both material and time costs. Therefore, the authors propose the use of a combination of computer and physical modelling, in order to properly select the process parameters of the hot forging process and the shape of the working surfaces of the dies, to ensure the proper quality of the forgings.

## 2. Purpose of this Work

The aim of this work was to analyze the effect of the elongation of magnesium alloy AZ91 in flat dies on the welding of internal metallurgical discontinuities. Model discontinuities, specifically of the metallurgical void type, were located in various local zones of the modeled rod to check the influence of their location on their welding. The behavior of the central porosity-type discontinuity located in the axis of the numerically modeled rod during the elongation operation was also studied.

The analysis of the welding of metallurgical discontinuities was carried out for two different geometrical shapes of the feedstock, namely, a cylinder and a cuboid. The aim was to check to what extent the geometric shape of the input material affects the welding of discontinuities’ volume during the elongation operation. The cylindrical- and cuboid-shaped samples were elongated, and all of the operation parameters were identical.

The numerical analysis was validated by physical tests in the Gleeble 3800 metallurgical process simulator (Dynamic System Inc., Poestenkill, NY, USA), using the same die shapes and main parameters of the elongation operation. This made it possible to check to what extent the results obtained during the numerical modeling in the FORGE^®^NxT 2.1 application coincided with the results of the physical modeling. In addition, an analysis of the structure of the magnesium alloy after elongation was carried out in places where there were modeled discontinuities and in places where there were no such discontinuities, and the resulting structures were compared.

The physical tests aimed to verify the results obtained from the numerical simulations, as well as to check whether, after the elongation operation, there were cracks in the reforged material in places of discontinuity, as well as in their external zones.

## 3. Material Used for this Research

The material chosen for the tests was magnesium alloy AZ91 with chemical compositions as given in Table 1.

## 4. Methodology of Numerical Research

**Figure 1:**
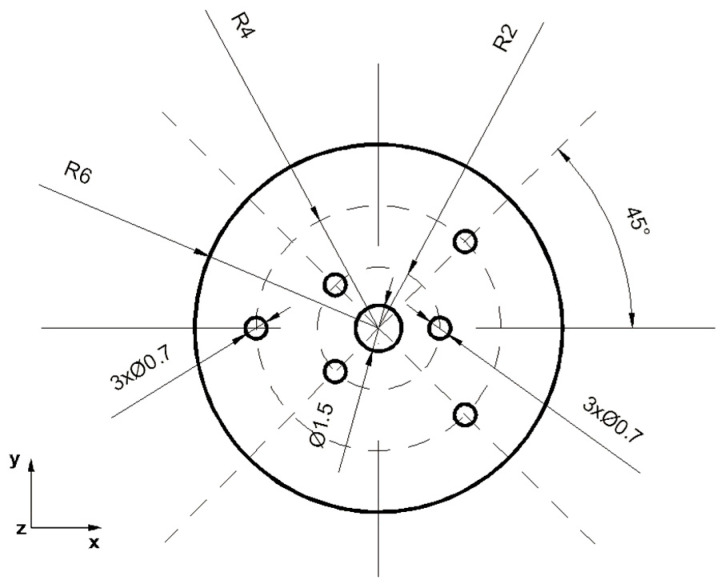
Front view of the cylinder-shaped model feedstock with the dimensions and locations of the introduced metallurgical discontinuities.

**Figure 2:**
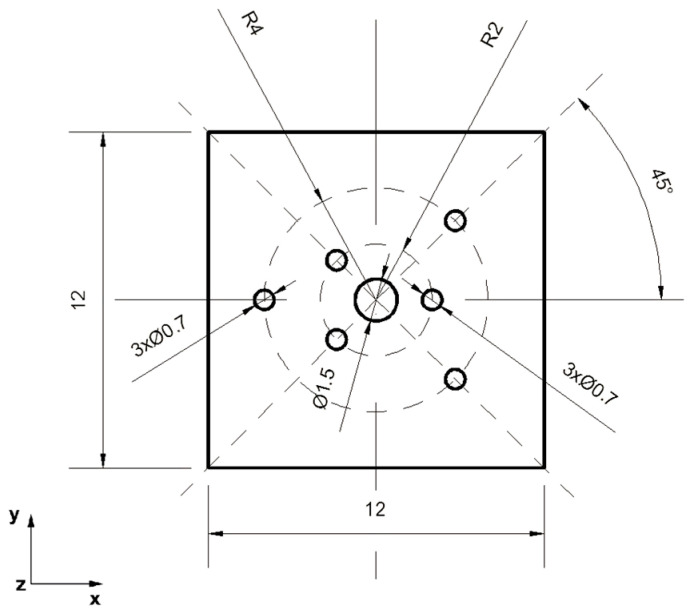
Front view of the cuboid-shaped model feedstock with the dimensions and locations of the introduced metallurgical discontinuities.

**Figure 3:**
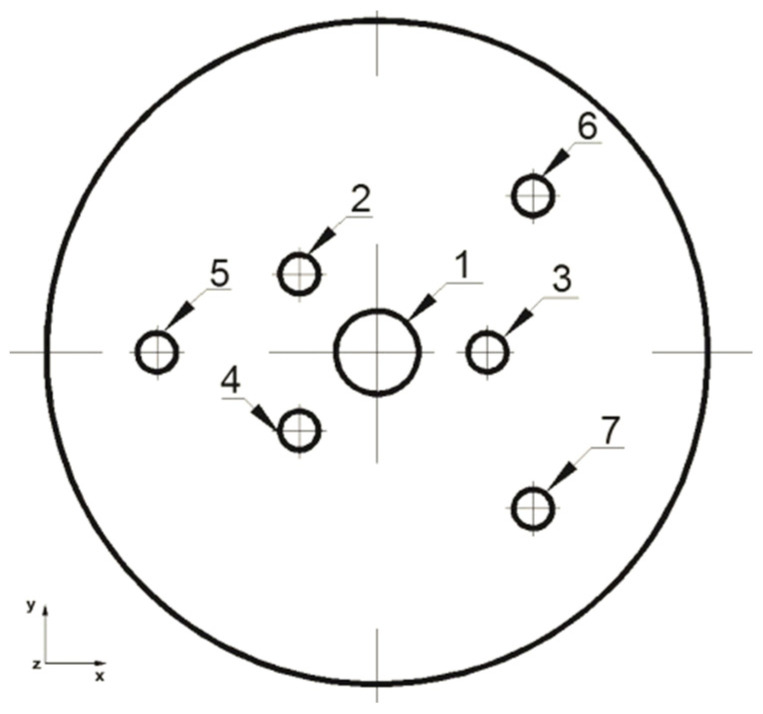
Numbering of the discontinuities in front view after 90° clockwise rotation (cylindrical feedstock).

**Figure 4:**
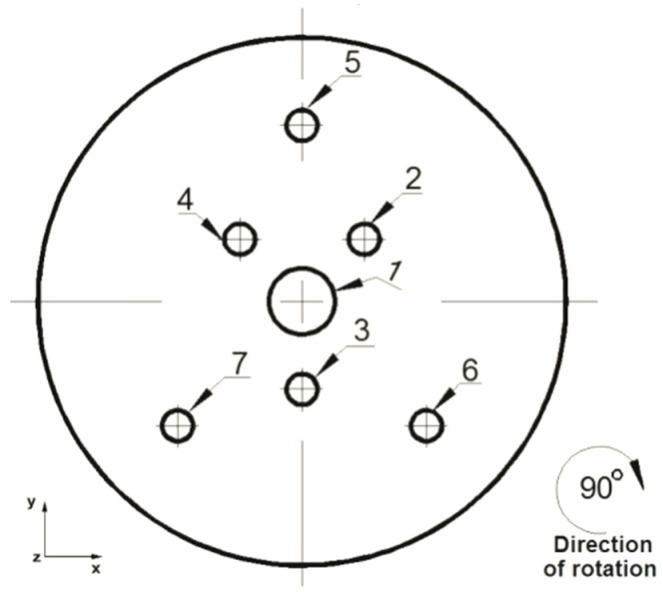
Numbering of the discontinuities in front view after 90° clockwise rotation (cylindrical feedstock).

**Figure 5:**
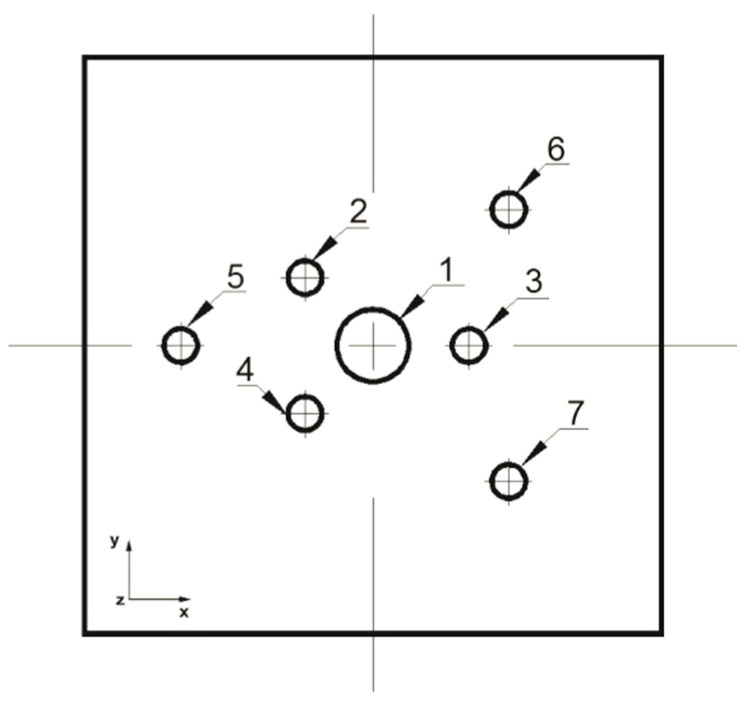
Numbering of the discontinuities in front view before 90° clockwise rotation (cuboid feedstock).

**Figure 6:**
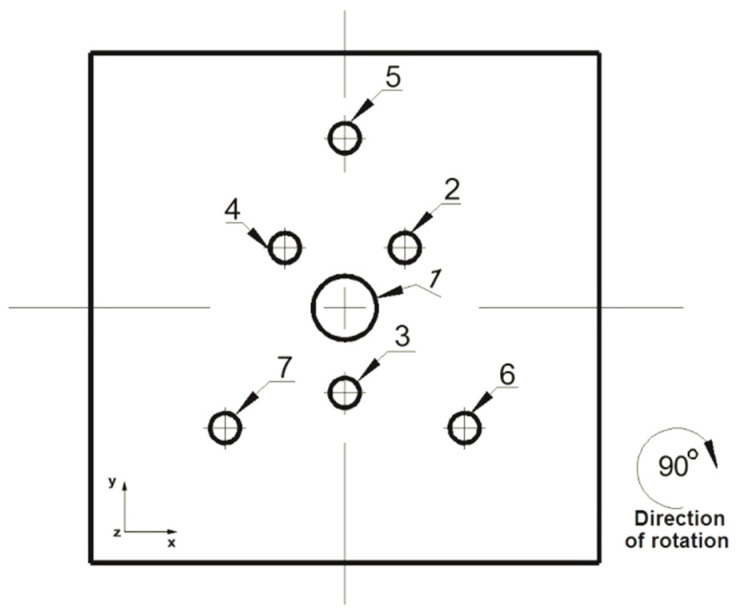
Numbering of the discontinuities in front view before 90° clockwise rotation (cuboid feedstock).

The analysis of the elongation operation in flat dies was carried out in the commercial computer application FORGE^®^NxT 2.1 based on the finite element method, for two model samples made of magnesium alloy AZ91, one in the shape of a cylinder with a diameter of 12 mm and a length of 27 mm (Figure 1) and the other in the shape of a cuboid with dimensions 12 × 12 mm and a length of 27 mm (Figure 2). Internal metallurgical discontinuities were modeled for both samples. Axial model discontinuities marked with a number 1, with a diameter of 1.5 mm, were to simulate metallurgical discontinuities of the central porosity type [31]. Model discontinuities marked with numbers 2–7, with a diameter of 0.7 mm, were to simulate internal discontinuities of the metallurgical void type [31]. The diameters and distribution of the individual model discontinuities were selected by the authors in accordance with the theory of casting processes [21,23,31]. The discontinuities were made in the model feedstock in the form of cylinders with a length equal to the length of the model feedstock, i.e., 27 mm (throughout). For the purposes of the numerical modeling, the cylindrical and cuboid models were drawn in the commercial computer application AutoCad 2009^®^, and the simulated discontinuities were treated as a difference in volume between the model feedstock and the simulated discontinuity. Figure 1 shows the arrangement and dimensions of the simulated discontinuities for the model cylinder-shaped feedstock and Figure 2 shows the same for the model cuboid-shaped feedstock. The numbering of the defects before the first and second deformation, for the individual shapes of the model feedstock, is presented in Figure 3, Figure 4, Figure 5 and Figure 6. The numbering of the simulated discontinuities was added to facilitate the analysis of results of the numerical modeling and their subsequent description.

The commercial computer program FORGE^®^NxT 2.1 from Transvalor Solution was used to model the elongation operation of the model magnesium alloy samples. This program allows for thermomechanical simulation of plastic working processes, among others [32]. A detailed description of the temperature, energy, stress, and strain functionals, as well as the thermomechanical and friction laws that are used during calculations, can be found in [33]. The hardening curves [17] were approximated using the Hensel–Spittel equation [34], which is embedded in this program. The program is based on the finite element method (FEM). A thermo-viscoplastic model of a deformed body, based on the theory of large plastic strains, was used to simulate the elongation operation. Tetrahedral elements with triangle bases were used to generate the finite element mesh. The value of the friction coefficient between the surfaces of the dies and the deformed rod was equal to μ = 0.3. The heat transfer coefficient between the dies and the material was assumed to be λ = 10,000 W/m^2^K, while the heat transfer coefficient between the magnesium alloy and the environment was assumed to be α = 10 W/m^2^K. The ambient temperature was assumed to be equal to 20 °C and the temperature of the dies was assumed to be equal to 300 °C. The initial temperature of the model feedstock before deformation in its entire volume was assumed to be the same, i.e., equal to 400 °C. The cylindrical- and cuboid-shaped models were deformed with a relative draft equal to 35%. The feed rate of the upper die was equal to v = 8 mm/s, while the lower die was assumed to be stationary. During the numerical modeling, both models (Figure 1 and Figure 2) were deformed with flat dies with a relative draft of 35%, then they were rotated clockwise by an angle of 90° and deformed again with the draft of the same value.

## 5. Research Methodology in the Gleeble 3800 Physical Process Simulator

**Figure 7:**
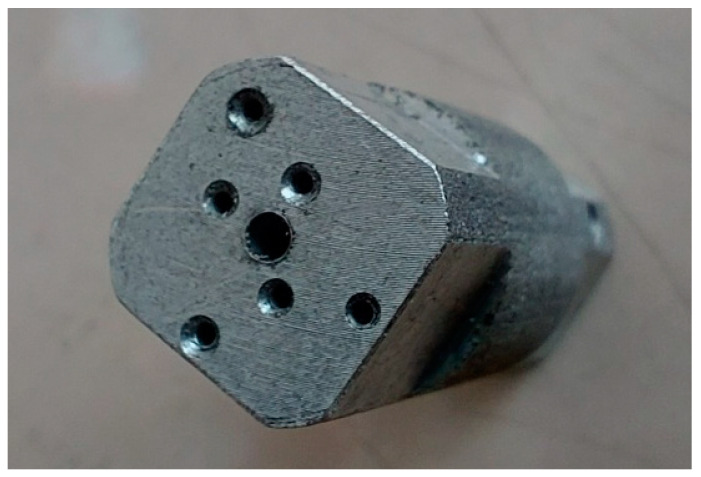
The square sample cross-section of 10 × 10 mm and a circular cross-section with a diameter of 12 mm prepared for the deformation process in the MaxStrain device.

In order to verify the numerical tests of the elongation process of the material with the artificially introduced discontinuities, physical simulations were performed using the MaxStrain module of the Gleeble 3800 system. Two types of samples were used for the tests including a square cross-section of 10 × 10 mm and a circular cross-section with a diameter of 12 mm. Holes were made in both samples as shown in Figure 1 and Figure 2. The holes were a model image of metallurgical discontinuities appearing in the cast feedstock material intended for the elongation operation. Figure 7 shows the sample prepared for deformation.

The samples were heated to 400 °C, held at this temperature for 150 s, and then deformed in flat dies with a 35% draft, reducing the initial height from 10 mm to 6.5 mm for the square sample, and from 12 mm to 7.8 mm for the round sample. After the deformation, the samples were heated to 400 °C, rotated by an angle of 90°, and another deformation was applied in such a way to obtain samples with final heights of 10 mm (for the square sample) and 12 mm (for the round sample) from the material expanded after the first deformation. A constant travel speed of the dies of 8 mm/s was used in both cases.

Table 2 provides a schematic of the elongation operations used in the numerical tests as well as during the verification of these tests under laboratory conditions.

## 6. Analysis of Numerical Research Results

### 6.1. Analysis of Temperature Distributions during the Elongation Operation of the Round Sample

**Figure 8:**
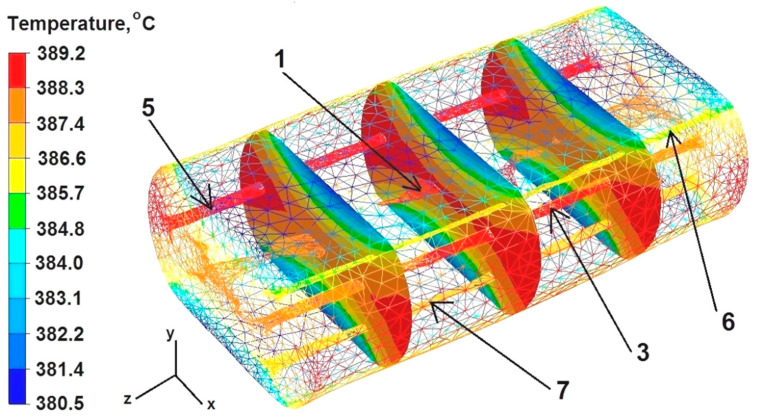
Distribution of temperature values after the first deformation of the round sample.

The data presented in Figure 8 show that the temperature value ranged from 389 to 386 °C in the middle part of the sample along the Z axis. On the right and left sides of the sample, along the X axis, the temperature was 389 °C because the freely flowing metal under the pressure from the upper die did not cool down—it was not limited by the working surface of the dies and only gave off heat to the environment.

Although the dies were heated to 300 °C before the deformation of the sample, the deformed sample was cooled in the metal–die contact zones by transferring heat towards the working surfaces of the flat dies. The sample temperature in these zones was in the range of 380 to 383 °C.

The data presented in Figure 8 show that discontinuities number 2 and 4 were completely welded after the first deformation. Discontinuity number 1 was significantly leveled, but not completely welded, even though the temperature in the axis of the deformed rod was 389 °C. Discontinuities number 5 and 3, despite maintaining very high temperatures (discontinuity 3 maintained this especially in its central part), remained practically unclosed. This is related to the location of these discontinuities in the X and Y symmetry axes of the sample. Discontinuities 7 and 6 were somewhat deformed, although their temperature was about 385 °C. These discontinuities were not located in the symmetry axes of the deformed sample, but within a radius of 4 mm from the Z axis. On this basis, it can be concluded that the welding of discontinuities is more influenced by their location—the distance from the Z axis—than by temperature in the area of the discontinuity being welded. The closer the discontinuities are to the Z axis of the sample, the more they weld during the first deformation, as in the case of discontinuities number 2 and 4. On the other hand, the discontinuities located near the generatrix of the cylinder are not even partially welded. Also, the discontinuities located in the symmetry axes of the forged sample (X and Y) are not welded, regardless of whether they are located near the Z axis or not (discontinuities 3 and 5). Therefore, in order to weld discontinuities located in the X and Y axes, it is recommended to forge the feedstock, rotating it during successive deformations not by an angle of 90°, but by an angle of 45°, or to use shaped and flat dies, not only flat ones.

**Figure 9:**
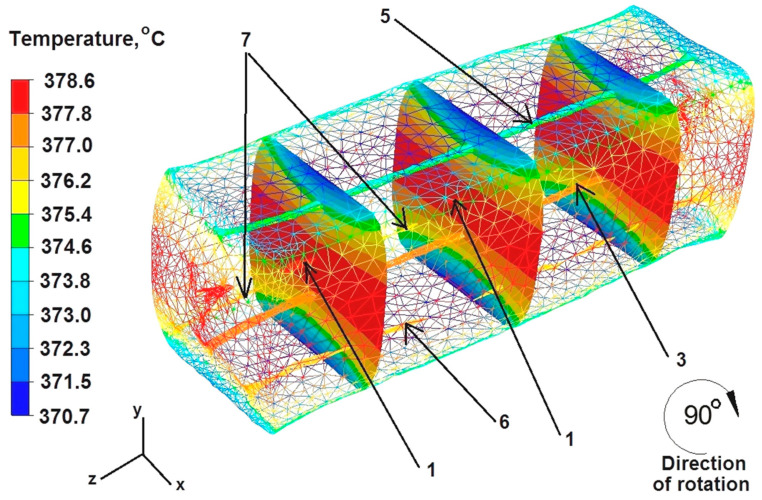
Distribution of the temperature values after the second deformation of the round sample.

Upon analyzing the data in Figure 9, it can be concluded that after the second deformation of the sample previously rotated by an angle of 90° in its axial zone, the temperature was about 378 °C. On the right and left sides of the sample, along the X axis, the temperature was also 378 °C. The temperature in the sample–die zone ranged from 374 °C to 370 °C. After the second deformation, discontinuity number 1 was further welded, but it was not welded completely, which is marked with arrows in Figure 8. Discontinuities 5 and 3 were not welded, due to the fact that after the rotation of the sample by 90° they were again located in the axes of symmetry X and Y. Their shape resembles the input state before the deformation. The temperature within discontinuity number 3 was 377 °C and within discontinuity number 5 it was 373 °C. Discontinuities 6 and 7 did not fuse completely because, as the authors suppose, they were located at a certain distance from the axis of the model sample. The temperature within discontinuities 6 and 7 was 376 °C.

### 6.2. Analysis of Effective Strain Distributions during the Elongation Operation of the Round Sample

**Figure 10:**
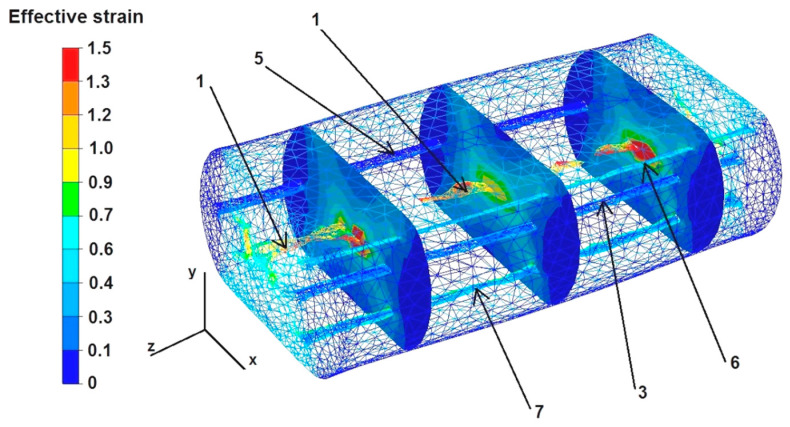
Distribution of effective strain values after the first deformation of the round sample.

The data in Figure 10 show that the deformation intensity values are highest in the middle part of the sample after the first deformation and range from 0.6 to 1.5. In the zones of free metal flow on the right and left sides of the sample, along the X axis, where the metal flow was not limited by the working surfaces of the flat dies, the deformation intensity values were small and ranged from 0.1 to 0.3. The maximum value of strain intensity was in the axial zone of the deformed sample along the Z axis and was 1.5. However, this high value of strain intensity did not result in the complete welding of discontinuity number 1. A discontinuity diameter that was too large was probably the reason for this. Discontinuities number 2 and 3, located 2 mm away from the Z axis of the rod, i.e., in the zone of high deformation intensity values in the range of 1.2–0.9, but smaller in diameter, were already welded after the first deformation. However, the locations of the discontinuities in the zone of large deformations were not the only sufficient condition for their complete welding. As the results from the analysis of the data presented in Figure 9 show, an additional condition for their complete welding must be that the location of the discontinuity is outside of the symmetry axes X and Y of the forged sample, preferably at an angle of 45° to these axes. Discontinuity number 3 did not weld, despite being located in the area of large deformations and high temperature, which was favorable for the welding process (Figure 7). Discontinuity number 5 was also not welded because it was located at a considerable distance (4 mm) from the zone of large deformations. The strain intensity values in this zone ranged from 0.1 to 0.3. Discontinuities 6 and 7 were inclined at an angle of 45° to the axes of symmetry X and Y, and also spaced 4 mm from the Z axis of the sample, but in this zone a higher value of strain intensity occurred, which led to the partial welding of these discontinuities. Based on the analysis of the test results, it can be concluded that the strain intensity values in the zone located at a distance of 2 mm from the Z axis of the sample were sufficient to completely weld the discontinuities, while the strain intensity values in the zone 4 mm away from the Z axis of the sample were too small to fully weld the metallurgical discontinuities.

**Figure 11:**
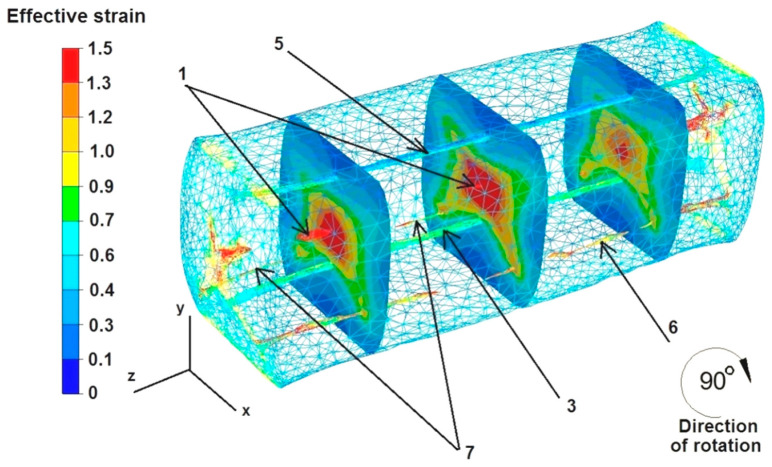
Distribution of effective strain values after the second deformation of the round sample.

The data presented in Figure 11 show that after the second deformation of the sample, previously rotated by an angle of 90°, the value of the strain intensity in its axial zone did not increase and amounted to 1.5, while in the remaining area it was in the range of 1.2–0.7. Discontinuities 5 and 3 were still not welded after the second deformation because they were located in the X and Y axes of the sample. Discontinuities 6 and 7 were also not welded, although they were located at an angle of 45° to the X and Y symmetry axes and in the zone where the strain intensity values were in the range of 1.5–0.9. This is probably related to the free flow of metal not constrained by the working surfaces of the dies. Discontinuity 1 also did not fully weld.

### 6.3. Analysis of Hydrostatic Pressure Distributions during the Elongation Operation of the Round Sample

**Figure 12:**
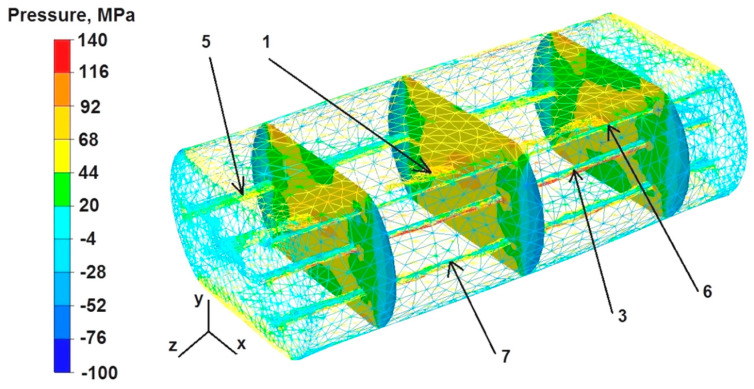
Distribution of pressure values during the first deformation of the round sample.

The data in Figure 12 show that the hydrostatic pressure values in the middle part of the sample after the first deformation ranged from 116 to 44 MPa. Hydrostatic pressure did not exist on the outer zones of free metal flow on the right and left sides along the X axis of the deformed sample. Discontinuities 2 and 4 welded completely because they were in the zone of high compressive stresses. The hydrostatic pressure values ranged from 92 to 68 MPa. Discontinuities 6 and 7, located 2 mm away from the Z axis, were not fully welded because the values of compressive stresses in this area were lower and ranged from 44–20 MPa. On this basis, it can be concluded that for closing discontinuities it is advantageous to maintain compressive stresses with values greater than 60 MPa. On the other hand, hydrostatic pressure values lower by 50 MPa do not have a positive effect on the process of welding internal metallurgical discontinuities. The second unfavorable factor is the location of the discontinuities in the symmetry axes (X and Y) of the deformed bar sample, regardless of the deformation value. Examples of this include discontinuities 3 and 5, which did not weld despite two successive deformations. Discontinuity 1 was not completely welded, despite being located in the axis of the deformed sample, where the value of hydrostatic pressure was the maximum and amounted to 116 MPa.

**Figure 13:**
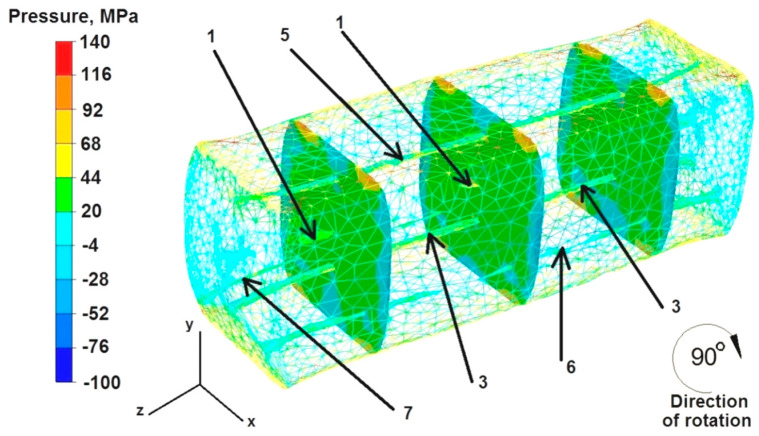
Distribution of pressure values during the second deformation of the round sample.

Based on the data presented in Figure 13, it can be concluded that after the second deformation of the sample previously rotated by 90°, practically in the entire volume, the value of hydrostatic pressure was uniform and amounted to 44 MPa. The only exceptions were the outer zones of the sample on the right and left sides along the X axis, where hydrostatic pressure did not exist (omnidirectional stretching occurred there). Hydrostatic pressure also did not occur at the point of contact between the forged sample and the working surfaces of the dies. However, the compressive stress of 44 MPa, which occurred practically in the entire volume of the deformed sample, was not sufficient to completely weld discontinuities 1, 7, and 6. Discontinuities 3 and 5, located in the Y axis after rotating the sample by 90°, were not welded. On this basis, it can be concluded that samples of the geometric shape of a cylinder should be rotated between successive pressures of the dies during the operation of forging elongation by an angle of 45°, and not, as was the case during the numerical modeling, by the angle of 90°.

### 6.4. Analysis of Temperature Distributions during the Elongation Operation of the Square Sample

**Figure 14:**
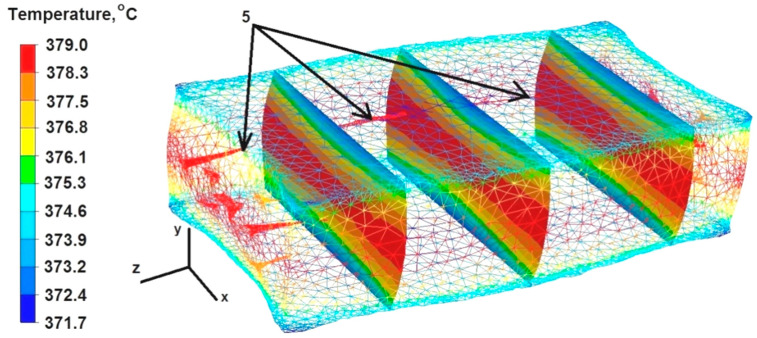
Distribution of temperature values after the first deformation of the square sample.

The data presented in Figure 14 show that the temperature value ranged from 379 °C to 376 °C in the middle part of the sample along the Z axis, while the right and left sides of the sample along the X axis, where the freely flowing metal did not cool down under the pressure from the upper die, maintained a temperature of 379 °C.

Although the dies were heated to 300 °C before the deformation of the sample, the deformed sample was cooled down in the metal–die contact zones by giving off heat towards the working surfaces of the flat dies. The temperature of the bar in these zones was in the range of 374–371 °C.

After the first deformation of the cuboid sample, all the discontinuities were welded except for discontinuity number 5 (Figure 14). Although the temperature within the discontinuity was the maximum, the distance of this discontinuity from the zone of large deformations was significant and, additionally, it was located in the X axis. The location of the discontinuities in the symmetry axes of the sample, regardless of its geometric shape, is not conducive to the process of discontinuity welding. The not fully welded discontinuity number 5 is marked with arrows in Figure 13. Based on the comparison of the test results obtained after the first deformation of the cylindrical sample (Figure 7) with the results obtained after the first deformation of the cuboid sample (Figure 14), it can be concluded that the geometric shape of the feedstock material has a large impact on the welding of discontinuities. The cylindrical and cuboid shapes were selected for numerical modeling because these forms are most often used in forging practice, as well as in other plastic working processes of metals and alloys. It is worth noting that the numerical modeling of both geometric shapes was carried out assuming the same process parameters for the elongation operation, so it is clearly visible that the geometric shape of the feedstock had a decisive influence on the welding of internal metallurgical discontinuities. Only two discontinuities were closed in the cylinder-shaped sample, and all discontinuities were closed in the rectangular sample except for discontinuity 5.

**Figure 15:**
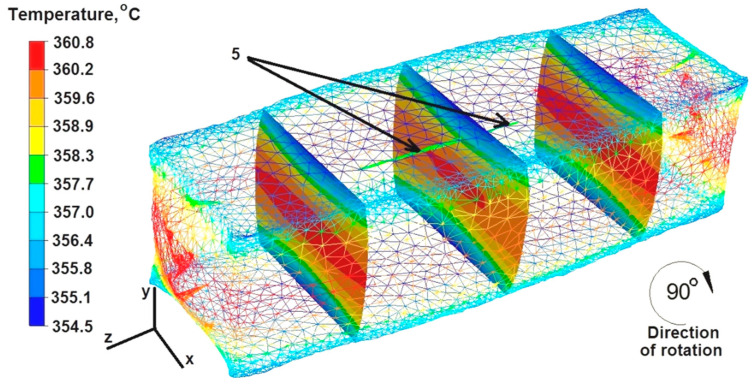
Distribution of temperature values after the second deformation of the square sample.

Based on the data in Figure 15, it can be concluded that after the second deformation of the sample, previously rotated by an angle of 90°, the temperature ranged between 360 °C and 357 °C in its central part and on the right and left sides along the X axis. However, in the lower and upper zones of material–die contact, the temperature was about 355 °C. Although the temperature around the welded discontinuity was 358 °C, it did not lead to the complete welding of discontinuity number 5, which is marked with arrows in Figure 14.

### 6.5. Analysis of Effective Strain Distributions during the Elongation Operation of the Square Sample

**Figure 16:**
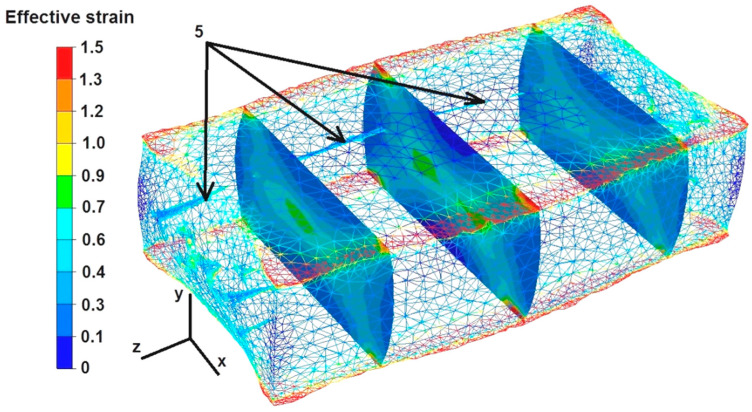
Distribution of effective strain values after the first deformation of the square sample.

The data in Figure 16 show that the strain intensity values were small in the middle part of the sample along the axis Z, ranging from 0.4 to 0.6. However, the strain intensity values increased slightly to 0.9 in the cross-sections shown in the figure. All the discontinuities except for discontinuity number 5 were completely welded, despite such a small growth of the strain values inside the deformed sample. The strain intensity values in the zone of not fully welded discontinuity number 5 ranged from 0.1 to 0.6. The maximum strain intensity values at the edges of the rectangular sample ranged from 1.3 to 1.5.

**Figure 17:**
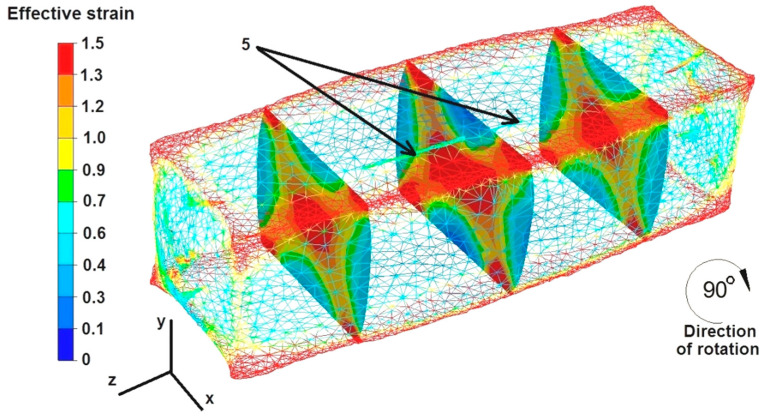
Distribution of effective strain values after the second deformation of the square sample.

Based on the data in Figure 17, it can be concluded that after the second deformation of the sample, previously rotated by 90°, the intensity of the deformation was much greater than in the case shown in Figure 16. The maximum strain intensity values occur not only at the edges of the sample but are also visible in all the cross-sections at the forging cross, and are very characteristic, especially during the deformation of metals in flat dies. The values of strain intensity in the forging cross ranged from 1.0 to 1.5. The values of strain intensity outside the forging cross, i.e., in the zone of high values of friction forces that inhibited the free flow of metal along the X axis in both directions, ranged from 0.1 to 0.6. Also, outside the forging cross, in the places of free metal flow not limited by the working surfaces of the dies, i.e., on the right and left sides of the X axis, there were tensile deformations of 0.1–0.6. The second deformation did not completely weld discontinuity number 5. The strain intensity values around this discontinuity ranged from 0.7 to 0.9.

### 6.6. Analysis of Hydrostatic Pressure Distributions during the Elongation Operation of the Square Sample

**Figure 18:**
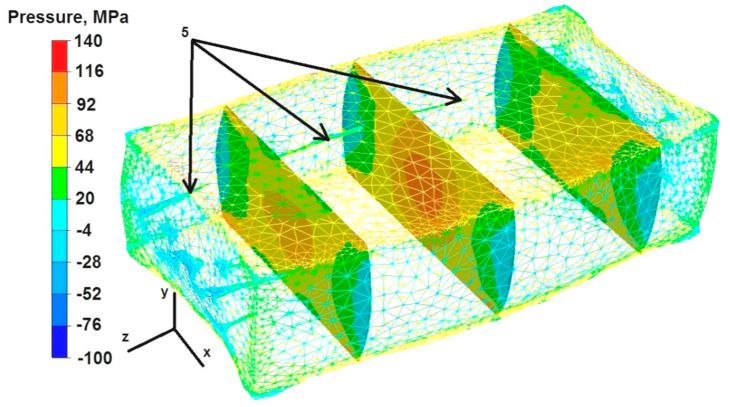
Distribution of pressure values during the first deformation of the square sample.

The data in Figure 18 show that the values of hydrostatic pressure ranged from 44 to 116 MPa in the predominant area of the cross-sections during the first deformation of the sample. The only exceptions were the zones located in the cross-sections on the right and left side of the deformed sample, where the unrestricted material flowed freely as a result of the draft. The pressure values there were low or null, within the range of −28 MPa to 20 MPa, which means that there were also tensile stresses. The hydrostatic pressure values in the zone of welded discontinuity number 5 were not the maximum and ranged from 20 MPa to 44 MPa.

**Figure 19:**
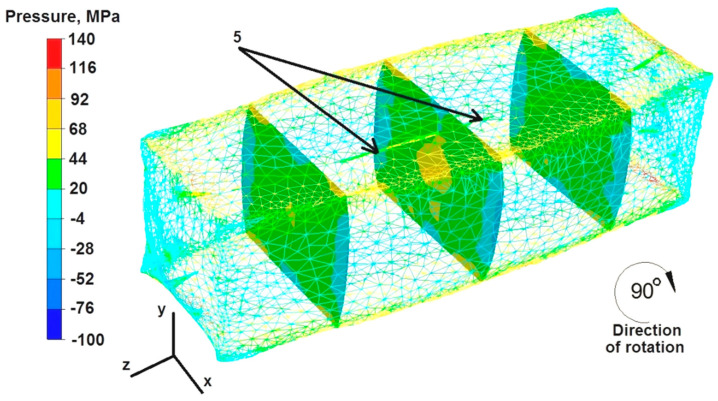
Distribution of pressure values during the second deformation of the square sample.

Based on the data presented in Figure 19, it can be concluded that during the second deformation of the sample, previously rotated by 90°, the distribution of hydrostatic pressure values in almost the entire analyzed area was smaller than during the first deformation (Figure 18) and ranged from 44 MPa to 68 MPa. The hydrostatic pressure outside this area ranged from −4 MPa to −28 MPa, which means that there was no hydrostatic pressure there, only tensile stresses. In the zone of welded discontinuity number 5, during the second deformation, the hydrostatic pressure values were 44–68 MPa.

## 7. Physical Simulation Results

**Figure 20:**
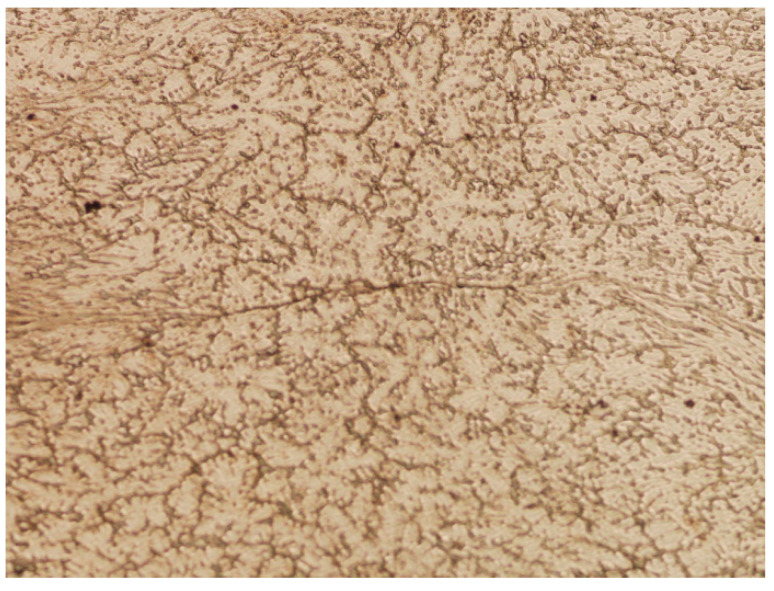
Closed axial discontinuity No. 1 in the deformed square sample (magnification 5×).

**Figure 21:**
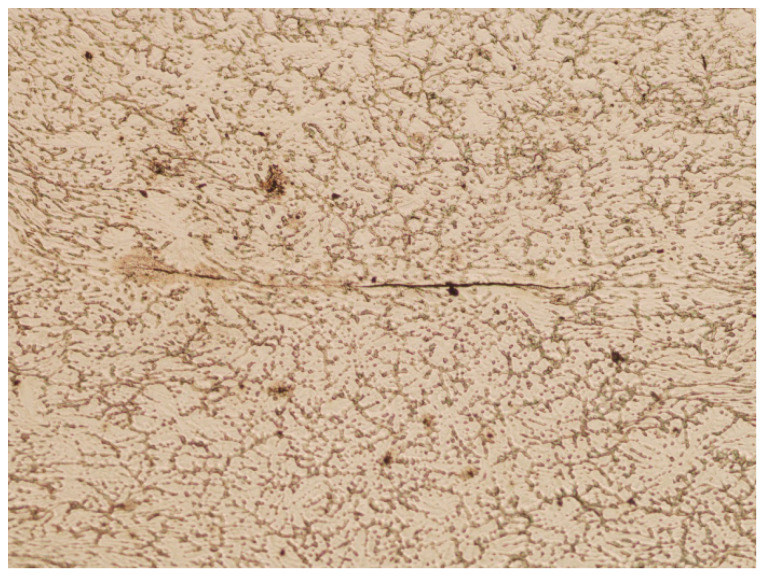
Partially closed axial discontinuity No. 1 in the deformed round sample (magnification 5×).

The tests carried out using the MaxStrain device made it possible to verify the numerical tests by analyzing the shape of the post-deformation traces of the discontinuities artificially introduced into the material. The samples subjected to the physical simulation of forging were cut in a plane perpendicular to their axes in half of their lengths. Metallographic microsections were made and microscopic images of the areas where the discontinuities were introduced were analyzed. In the following description, the same discontinuity numbering was used as during the numerical modeling of the process. Figure 20 and Figure 21 show the areas of axial discontinuity of the samples after deformation of the square and round samples, respectively.

In Figure 20, in the middle part, the resulting boundary after closing the axial discontinuity in the square sample is visible. No voids were found in this area, which means that the deformation conditions favored the complete closure of the artificially introduced discontinuity.

On the other hand, in Figure 21, in the middle part, the partially closed axial discontinuity No. 1 is visible in the round sample. In this case, gaps were found at the ends of the line formed by the closure of the void. Thus, under the assumed deformation conditions, it cannot be claimed that the axial metallurgical void was closed.

**Figure 22:**
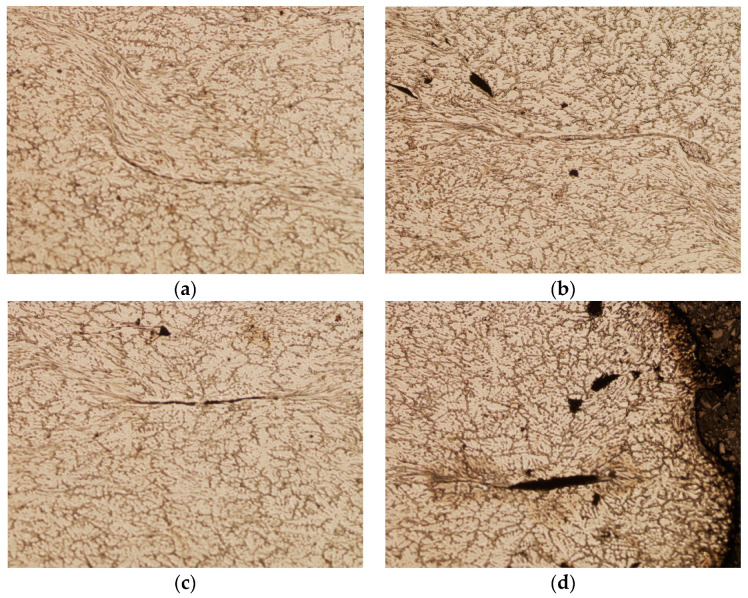
Areas of material identified in the square sample after deformation, corresponding to the locations of the artificially introduced discontinuities in accordance with the numbering 2 (**a**), 7 (**b**), 3 (**c**), and 5 (**d**) (magnification 5×).

Figure 22 presents images of metallographic microsections made on the intersection surface of the square sample after deformation, corresponding to the areas of the artificially introduced discontinuities. The holes marked with numbers 2 and 7 were located on the line determined by the diagonal of the sample cross-section before the deformation and were located at a distance of 2 mm and 4 mm from the axis of the sample, respectively. On the other hand, holes numbered 3 and 5 were placed on the horizontal line in the middle of the sample height at 2 mm and 4 mm away from the axis of the sample, respectively. The observations of the areas revealed on the microsections showed that the discontinuities marked with numbers 2 and 7 were fully closed after the deformation, which corresponds to the results of the numerical simulation. Figure 22a,b show the lines formed after the voids were closed, but no separation of the material was found in their surroundings. The areas where holes 3 and 5 were located look different. In this case, in the area of discontinuity No. 3, located closer to the axis, a line was found resulting from its closure, but a more thorough assessment of this line allowed us to conclude that there were areas not completely closed along its length. The hole marked with number 5, located 4 mm from the axis of the sample, remained largely open. It should be noted that in the case of the artificial defect marked with number 3, full compliance with the prediction based on the numerical simulation was not obtained.

**Figure 23:**
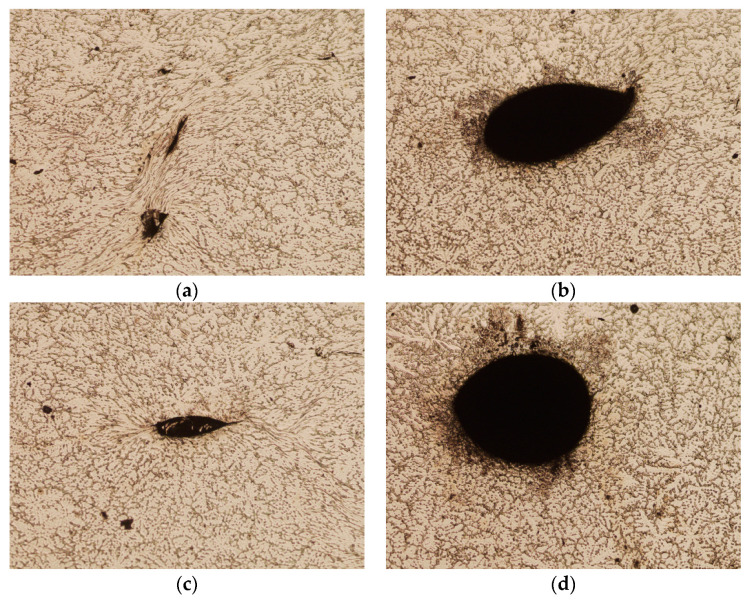
Areas of material identified in the round sample after the deformation, corresponding to the locations of the artificially introduced discontinuities in accordance with the numbering 2 (**a**), 7 (**b**), 3 (**c**), and 5 (**d**) (magnification 5×).

Figure 23 presents images of metallographic microsections made on the intersection surface of the round sample after deformation, corresponding to the areas of location of the artificially introduced discontinuities. As in the case of the square sample, the holes marked with numbers 2 and 7 were located, before deformation, on a line inclined at an angle of 45° relative to the plane of the dies and passing through the axis of the sample and were located at a distance of 2 mm and 4 mm from the axis of the sample, respectively. On the other hand, holes numbered 3 and 5 were placed on a horizontal line in the middle of the sample height at 2 mm and 4 mm away from the axis of the sample, respectively. The observations of these areas on the microsections showed that none of the analyzed discontinuities were fully closed after the deformation. The discontinuity marked with number 2 showed the greatest susceptibility to closure, which is similar to the results of the numerical simulation. In Figure 23a, the line formed after the partial closure of the void is visible, but incomplete closure of the material was found in part of its course. The areas where holes 7, 3, and 5 were located look completely different. None of the artificially introduced discontinuities were closed. Their deformation is visible—their ovalization occurred under the influence of plastic deformation of the material around them. The defect marked with number 3, shown in Figure 23c, was partially closed. Defect No. 7, shown in Figure 23b, was significantly ovalized, while the defect marked with No. 5 remained open, only slightly changing its shape, as shown in Figure 23d.

## 8. Comparison of Numerical and Physical Test Results

Table 3 summarizes the results of numerical tests and their verification under laboratory conditions on the effect of elongation operations on the welding of internal metallurgical discontinuities, for two different shapes of magnesium alloy bar.

Based on the data presented in Table 3, it was found that the physical modelling results confirmed the results obtained from the numerical modelling. Only in the case of the sample with a square cross-section does discontinuity No. 3 in the physical simulation appear incompletely closed (Figure 22c). The reason for this condition could be contamination of the hole that was drilled into the material to model the discontinuity. It should also be noted that, although a trace of the discontinuity is clearly visible, after the second deformation in the direction consistent with the length of the ‘gap’ formed from the discontinuity in the first deformation, it has not opened, indicating that the material has been welded in this area. In other cases, the numerical results were confirmed by laboratory tests.

Based on the analysis of the numerical results, it can be concluded that the geometric shape of the feedstock is of key importance in the process of welding metallurgical discontinuities, which was confirmed by the results of laboratory tests. The shape of the feedstock forces the flow kinematics of the metal into its deformed volume, which is why it is easier to weld discontinuities in a rectangular bar than in a cylindrical bar. In addition to the shape of the feedstock, the distance at which the discontinuities are located from the bar axis (Z axis) is also important. The closer to the bar axis that the discontinuities are welded, the more likely the discontinuities are to be completely welded during the first deformation, as in the case of discontinuities 2 and 4. Discontinuities lying in the division axes of the forged cylindrical bar (discontinuity 5) are particularly difficult to weld, especially if they are located in the zone of smaller deformations 4 mm away from the forged bar axis (Table 2, Figure 23d). Discontinuities located in the division axes of the forged bar (X and Y axes), regardless of the distance from the bar axis, as observed in discontinuity No. 3, are also not welded (Table 2, Figure 23c).

## 9. Conclusions

Based on the completed research, the following final conclusions were formulated:

1. In order to weld discontinuities, elongation operations should be carried out with the maximum possible relative deformation of 35%.
2. The geometric shape of the feedstock material and the locations of internal metallurgical discontinuities are of key importance in the process of discontinuity welding.
3. The kinematics of metal flow during the deformation of samples of various shapes is of particular importance in terms of the welding of metallurgical discontinuities (the shape of the feedstock material forces the kinematics of metal flow in the deformed volume).
4. It is easier to weld discontinuities in a cuboid-shaped material and it is much more difficult in a cylinder-shaped one.
5. Discontinuities are easier to weld when the deformation and stress values are high in the zones where they occur and amount to 1.2–1.5 and 96–116 MPa, respectively, while maintaining high temperatures close to the initial temperature around 400 °C in these zones.
6. High temperatures, around 400 °C, without high hydrostatic pressure and deformation intensity in the welded discontinuity zone is not a state sufficient for the complete welding of a discontinuity.
7. It is particularly difficult to weld discontinuities lying in the symmetry axes X and Y of the forged cylinder-shaped sample, especially those at a distance of 4 mm or more from the Z axis.
8. For a cylindrical feedstock, presumably a 45° rotation of the bar will produce more favorable results for welding discontinuities than a 90° rotation.
9. The analysis of the presented numerical modeling studies shows that in order to completely weld discontinuities in the free forging process, a sufficient range of hydrostatic pressure values is 96–116 MPa, and a sufficient range of deformation intensity values is 1.0–1.5 while maintaining a high temperature for the deformed sample.

## Figures and Tables

**Table 1:** Chemical composition of the investigated alloy (%).

Alloy	Zn	Al	Si	Cu	Mn	Fe	Ni	Mg
AZ91	0.59	8.98	0.05	0.006	0.23	0.013	0.003	R

**Table 2:** Elongation schemes for numerical and physical tests.

Number of Forging Passes	Square Sample		Round Sample	
	Relative Deformation, %	Angle of Rotation of the Bar, °	Relative Deformation, %	Angle of Rotation of the Bar, °
1	35	0	35	0
2	35	90	35	90

**Table 3:** Summary of numerical and physical test results.

Defect Number	Round Feedstock		Square Feedstock	
	Numerical Research	Laboratory Research	Numerical Research	Laboratory Research
1	Closed	Closed	Not closed	Not closed
2	Closed	Closed	Closed	Closed
3	Closed	Not closed	Not closed	Not closed
4	Closed	Closed	Closed	Closed
5	Not closed	Not closed	Not closed	Not closed
6	Closed	Closed	Not closed	Not closed
7	Closed	Closed	Not closed	Not closed

## Data Availability

Not applicable.
